# TIP48/Reptin and H2A.Z Requirement for Initiating Chromatin Remodeling in Estrogen-Activated Transcription

**DOI:** 10.1371/journal.pgen.1003387

**Published:** 2013-04-18

**Authors:** Mathieu Dalvai, Laurence Fleury, Luca Bellucci, Silvia Kocanova, Kerstin Bystricky

**Affiliations:** 1 Université de Toulouse, UPS, Laboratoire de Biologie Moléculaire Eucaryote (LBME), Toulouse, France; 2 CNRS, UMR5099, Toulouse, France; University of Cambridge, United Kingdom

## Abstract

Histone variants, including histone H2A.Z, are incorporated into specific genomic sites and participate in transcription regulation. The role of H2A.Z at these sites remains poorly characterized. Our study investigates changes in the chromatin environment at the *Cyclin D1* gene (*CCND1*) during transcriptional initiation in response to estradiol in estrogen receptor positive mammary tumour cells. We show that H2A.Z is present at the transcription start-site and downstream enhancer sequences of *CCND1* when the gene is poorly transcribed. Stimulation of *CCND1* expression required release of H2A.Z concomitantly from both these DNA elements. The AAA+ family members TIP48/reptin and the histone variant H2A.Z are required to remodel the chromatin environment at *CCND1* as a prerequisite for binding of the estrogen receptor (ERα) in the presence of hormone. TIP48 promotes acetylation and exchange of H2A.Z, which triggers a dissociation of the *CCND1* 3′ enhancer from the promoter, thereby releasing a repressive intragenic loop. This release then enables the estrogen receptor to bind to the *CCND1* promoter. Our findings provide new insight into the priming of chromatin required for transcription factor access to their target sequence. Dynamic release of gene loops could be a rapid means to remodel chromatin and to stimulate transcription in response to hormones.

## Author Summary

Our study investigates changes in the chromatin environment at the *Cyclin D1* gene that are a prerequisite for transcriptional initiation in response to estradiol. Gene expression is under control of chromatin structure. Histone variants, including histone H2A.Z, are incorporated into specific genomic sites and participate in transcription regulation. We show that H2A.Z is present at the transcription start-site and downstream enhancer sequences of *CCND1* when the gene is poorly transcribed. Stimulation of *CCND1* expression required release of H2A.Z concomitantly from both these DNA elements. The TIP48/reptin protein, which is part of several chromatin remodeling complexes, also associated with the *CCND1* regulatory elements. Here, TIP48 promotes exchange of H2A.Z, which triggers a dissociation of the *CCND1* enhancer from the promoter, thereby releasing a repressive intragenic loop. This release then enables estrogen receptor binding to the *CCND1* promoter. Acetylation of H2A.Z is required for these processes. Our findings provide new insight into the priming of chromatin required for transcription factor access to their target sequence. Hence, we propose a new model for early events in transcription activation that were not shown before. Specifically, release of looping could be a rapid means to activate transcription efficiently in response to stimuli, in particular estrogen.

## Introduction

Transcription activation relies on a choreography of local chromatin remodeling events that include posttranslational histone modifications and replacement of canonical histones by variants [1]–[4]. Chromatin immunoprecipitation (ChIP) studies have provided extensive information on the recruitment of these complexes by the hormone bound estrogen receptor in ERα-positive breast cancer cells [5]. The first complex to occupy promoter sequences is the ATP-dependent SWI/SNF chromatin remodeling complex and its catalytic subunit Brg1. Its activity enables subsequent binding of a plethora of histone and protein modifying assemblies which lead to transcription initiation by polymerase II [6]–[10]. In contrast, it is less clear how local chromatin structure prepares for rapid and massive recruitment of the estrogen receptor itself in the presence of estrogen.

Incorporation of histone variants constitutes a means to alter nucleosome properties and positioning at specific genomic loci. The histone H2A variant H2A.Z is frequently found within nucleosomes at regulatory sequences [11]–[14]. In particular, H2A.Z occupancy characterizes inducible and constitutive DNAseI hypersensitive sites to which nuclear receptors bind [15]. This variant is believed to induce a chromatin conformation that poises genes for transcription in human cells [16]. In yeast, H2A.Z exchange is mediated by the SWR1 complex [17], [18]. However, in mammalian cells, the mechanisms of H2A.Z deposition are still poorly characterized and may require several distinct protein complexes depending on the cellular context (for a review see [19]). Among ATP-dependent chromatin remodeling complexes the TIP48/TIP49 containing SWR1/SRCAP [20] or TIP60/p400 [21], [22] complexes have been shown to play a role in H2A.Z deposition [17], [18], [23]. p400 was also reported to be required for H2A.Z incorporation into the *TFF1*/*pS2* gene concomitant to estrogen receptor binding [24]. In an *in vitro* study Choi et al. demonstrated that the AAA+ family (ATPases Associated with various cellular Activities) members TIP48/TIP49 participate in the replacement of H2A by H2A.Z [25]. This H2A.Z exchange was facilitated by TIP60-mediated H2A acetylation. TIP48/TIP49 proteins (also known as TIP49b and TIP49a, Rvb2 and Rvb1, reptin and pontin) are important for assembly and activity of the histone TIP60 acetyltransferase complex [26].

To gain a better understanding of the early steps required in estrogen receptor mediated transcription activation and the coordination between remodeling complexes and chromatin structure, we analyzed transcription of the *cyclin D1* gene (*CCND1*) in ERα-positive MCF-7 breast cancer cells. This oncogene is frequently overexpressed in human breast tumors [27]. Its down-regulation increases migratory capacity and is linked to unfavorable prognosis [28], [29]. Cyclin D1 is a mitogenic sensor that modulates cell cycle progression. *CCND1* transcription is stimulated by 17β-estradiol (E2), inhibited by antiestrogens and cell cycle regulated in ERα-positive breast cancer cells [30], [31].

Here we show that TIP48 and H2A.Z associate with *CCND1* promoter and enhancer sequences. TIP48 is required for chromatin reorganization which is initiated by release of H2A.Z and opening of a repressive promoter-enhancer gene loop enabling TIP60 and the E2 bound estrogen receptor to be loaded to stimulate *CCND1* transcription.

## Results

### TIP48 and H2A.Z association with *CCND1* regulatory sites is required for activation by estradiol

**Figure 1:**
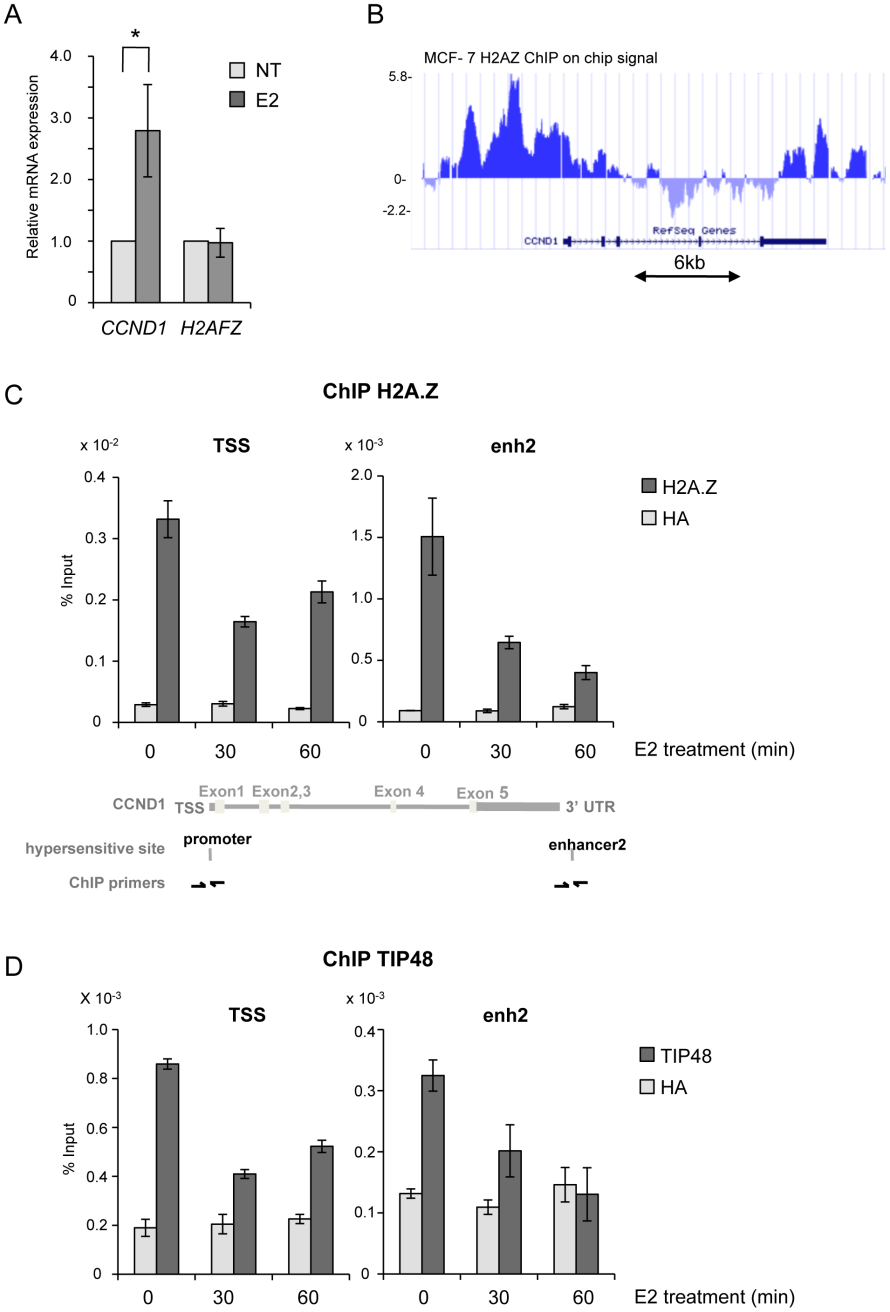
TIP48 and H2A.Z are required for *CCND1* activation by estradiol. A) MCF-7 cells were cultivated 3 days in steroid stripped (white) medium and then induced by E2 10^−7^ M for 6 h. *CCND1*, *H2AFZ* mRNA was quantified by real-time RT-PCR. The mean and SD from three independent experiments are shown. (*) indicates a p value<0.05 (Student t-test). B) MCF-7 H2A.Z ChIP on chip signal detected around the *CCND1* gene on human chromosome 11 loaded from [32] on UCSC. C, D) H2A.Z (C) and TIP48 (D) occupancy at *CCND1* TSS and enh2 before and after 30 min and 60 min of E2 10^−7^ M treatment analysed by ChIP. The values of ChIP efficiencies are given in % of input with s.e.m indicated (n = 3). Positions of DNAseI hypersensitive sites and primers used for ChIP are indicated in C).

**Figure 2:**
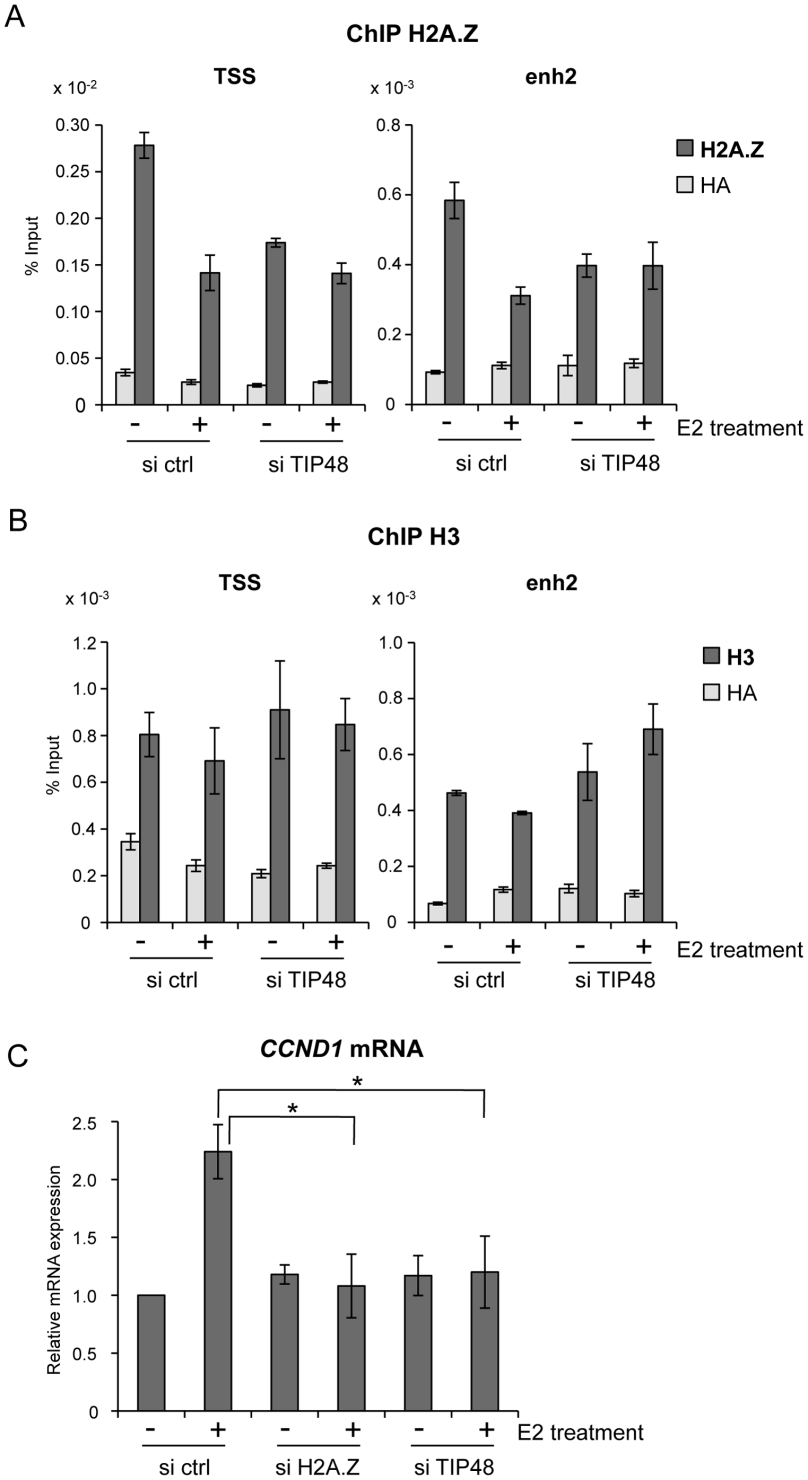
TIP48 regulates estrogen-activated transcription via H2A.Z release. A, B) MCF-7 cells were cultivated 3 days in steroid free medium, transfected with, scramble (ctrl) or TIP48 siRNA for 72 h and induced by E2 10^−7^ M for 30 min. Association of H2A.Z (A) and histone H3 (B) with TSS and enh2 sites was analyzed by ChIP and shown as % input (n = 2). C) MCF-7 cells were cultivated 3 days in steroid free medium and transfected at day 0 with a Scramble (Ctrl), a H2A.Z siRNA or a TIP48 siRNA for 72 h and then induced by E2 10^−7^ M for 6 h. *CCND1* mRNA levels were determined by qRT-PCR. Control (Ctrl) without E2 treatment was set to 1. The mean and SD from three independent experiments are shown. (*) indicates a p value<0.05.

In ERα-positive MCF-7 cells grown in steroid stripped media only basal transcription levels of the *Cyclin D1* gene (*CCND1*) were measured. Addition of E2 lead to a 2.5-fold increase in *CCND1* mRNA levels in cells treated 6 h with 100 nM E2 (Figure 1A). H2A.Z has been reported to act in concert with ER to regulate the *TFF1* gene [24] prompting us to examine H2A.Z association with *CCND1* regulatory elements. Analysis of ChIP-on-chip data revealed that H2A.Z was highly enriched at sequences 5′ and 3′ flanking the *CCND1* gene, and largely absent from the open reading frame (Figure 1B) [32]. By conventional ChIP, we found that the amount of H2A.Z present at the *CCND1* promoter was reduced by 50% at the TSS (Figure 1C). Eeckhoute *et al.* identified an enhancer (enh2) at the 3′ end of the *CCND1* gene which acts as the primary site for ERα and cofactor binding during *CCND1* transcriptional regulation [7]. We thus analyzed the chromatin organization of enh2. Similar to the promoter, H2A.Z present at enh2 was removed during transcription activation (Figure 1C). Reduced binding was not due to a decrease in *H2AFZ* expression in the presence of E2 (Figure 1A). Chromatin modifications at the *CCND1* promoter and enhancer appear to be coordinated. Removal of H2A.Z from promoter sequences upon transcription activation correlates with observations in yeast and several mammalian cells and points to a mechanism of regulation distinct from the one of the ERα target gene *TFF1* [33], [24], [34]. Replacement of nucleosomal H2A with H2A.Z has been shown to be catalyzed by the TIP48/49 complex *in vitro* [25]. The TIP48/49 complex was thus a good candidate for regulating H2A.Z dynamics at *CCND1* regulatory sequences. TIP48 and TIP49 are ubiquitously expressed and are often part of the same complex. In most cell types, and in particular in epithelial cancer cells such as MCF-7 cells, silencing one of the partners by interference RNA lead to degradation of the other partner [33]. Better antibody specificity and efficiency prompted us to investigate TIP48. TIP48 was associated with the promoter and enh2 of *CCND1* in non-induced ERα-positive MCF-7 cells (Figure 1D). Binding of TIP48 to the *CCND1* TSS and enh2 decreased rapidly following addition of 100 nM estradiol (E2). Expression of the gene coding for TIP48 was insensitive to E2 (Figure S1A). To examine the relationship between TIP48 and H2A.Z, we selectively depleted TIP48 by siRNA (Figure S1A). In siTIP48 transfected cells treated or not with E2, H2A.Z binding to *CCND1* was reduced compared to control, non–specific siRNA transfected cells. Levels of H2A.Z binding in the absence of TIP48 were roughly equivalent to levels in E2 treated control cells (Figure 2A). Moreover, nucleosome density assessed by immunoprecipitating histone H3 was unchanged near the TSS (Figure 2B). Thus, eviction of H2A.Z upon initiation of E2 stimulated transcription was not due to general chromatin decondensation around the *CCND1* gene and its promoter region in particular. TIP48 appears to be necessary for recruiting H2A.Z to the *CCND1* gene in MCF-7 mammary tumor cells.

We thus asked whether binding of H2A.Z or its release were important for regulating *CCND1* transcription. H2A.Z mRNA expression levels were reduced ∼5-fold 48 h post transfection with a smartpool siRNA directed against H2A.Z compared to control cells (Figure S1B). Reduced levels of H2A.Z did not alter basal *CCND1* expression levels, but impeded activation by E2 (Figure 2C). Similarly, in the absence of TIP48, basal transcription levels were conserved, while activation of *CCND1* by E2 was compromised (Figure 2C). *H2AFZ* mRNA levels were not affected by selective knockdown of TIP48 (Figure S2A). Thus, Stimulation of *CCND1* expression required release of H2A.Z concomitantly from both these DNA elements.

### TIP48 promotes ERα binding during E2 activated transcription

**Figure 3:**
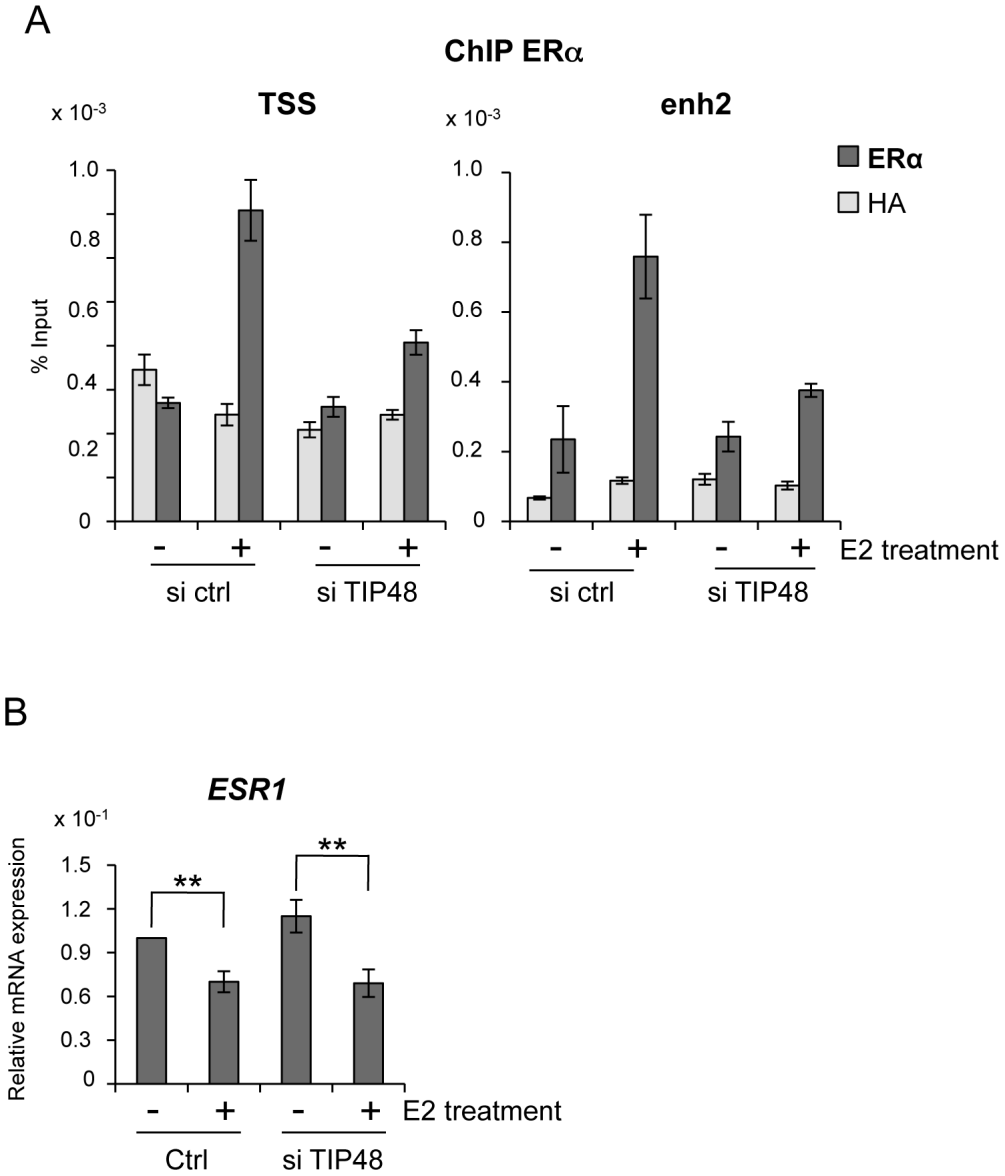
TIP48 is required for ERα binding during E2 activated transcription. MCF-7 cells were cultivated 3 days in steroid free medium, transfected with scramble (ctrl) or TIP48 siRNA for 72 h and induced by E2 10^−7^ M for 30 min. A) ChIP analysis of ERα occupancy at the *CCND1* TSS and enh2 sites. Results are shown as % input (n = 2). B) qRT-PCR quantification of *ESR1* mRNA expression relative to *RPLO*. The mean and SD from three independent experiments are shown. (**) indicates a p value<0.01.

Absence of activation was likely due to failure of ERα fixation to the *CCND1* promoter. Under standard conditions, E2 stimulated ERα binding to both the promoter and enh2 of *CCND1* (Figure 3A) [7]. Selective knock down of TIP48 hindered ERα binding to these sites (Figure 3A). Reduced binding could not be attributed to altered or decreased *ESR1* expression patterns in cells transfected with control or TIP48 siRNAs (Figure 3B). Therefore, TIP48 appears to be necessary to remodel *CCND1* chromatin structure for productive ERα binding in the presence of hormone.

### TIP48 promotes recruitment of TIP60 to *CCND1*

**Figure 4:**
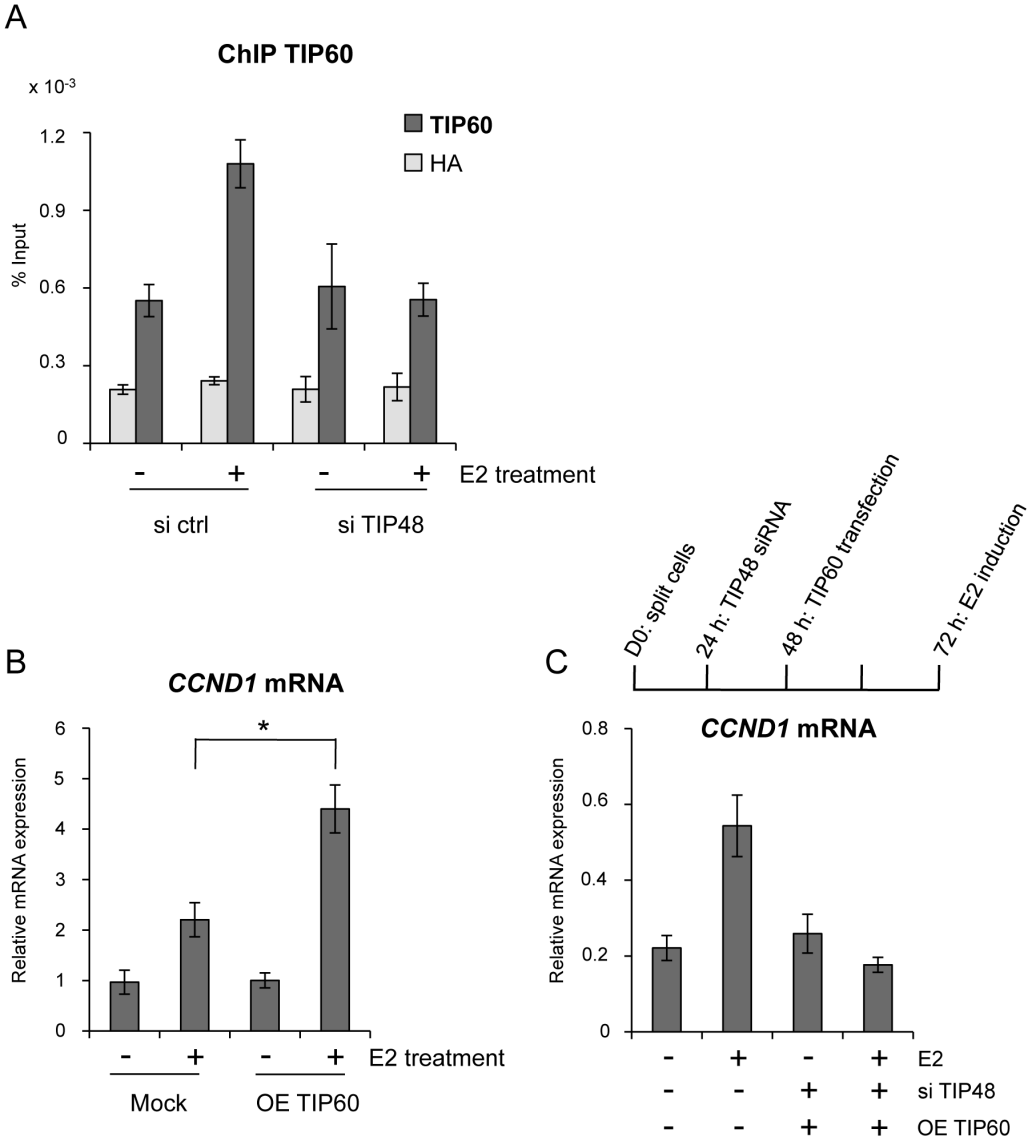
TIP48 promotes TIP60 binding and *CCND1* transcription activation. MCF-7 cells were cultivated 3 days in steroid free medium and induced by E2 10^−7^ M for 6 h. A) Cells were transfected with scramble (-) or TIP48 siRNA for 72 h and induced by E2 10^−7^ M for 30 min. TIP60 binding to the *CCND1* promoter was analyzed by ChIP and shown as percent of input (n = 2). B) After 24 h in steroid free medium, MCF-7 cells were transfected with a mock vector or a vector expressing TIP60 for 48 h. *CCND1* mRNA expression was analyzed by qRT-PCR. The mean and SD from three independent experiments are shown. (*) indicates a p value<0.05. C) MCF-7 cells were first transfected with scramble (-) or TIP48 siRNA as in (A). After 24 h, same cells were transfected with a mock vector or a vector expressing TIP60 for 48 h as in (B). *CCND1* gene expression was analyzed by qRT-PCR after 30 min of E2 10^−7^ M induction.

TIP48 and TIP60 have been found as part of the same complex [20], [25]. TIP60 also cooperates with ERα and other chromatin-remodeling enzymes during estrogen-induced transcription [34], [35]. We tested whether TIP48 and TIP60 binding to the *CCND1* promoter was coordinated. In the presence of E2, TIP60 was recruited to the *CCND1* promoter (Figure 4A). Upon depletion of TIP48, TIP60 no longer associated with the *CCND1* TSS (Figure 4A). Cooperation between TIP48, ERα and TIP60 binding was likely to be necessary for transcription activation. To unravel a functional link, we first over-expressed TIP60 in MCF-7 cells (Figure S2B). TIP60 overexpression stimulated E2 activated *CCND1* transcription nearly 5-fold compared to control untreated cells, without affecting neither basal, non-induced mRNA levels (Figure 4B) nor the expression pattern of the *H2AFZ* gene (Figure S2C). In siTIP48 transfected cells, overexpression of TIP60 was no longer able to stimulate *CCND1* transcription upon E2 stimulation (Figure 4C), suggesting that TIP48 is required for TIP60 function.

**Figure 5:**
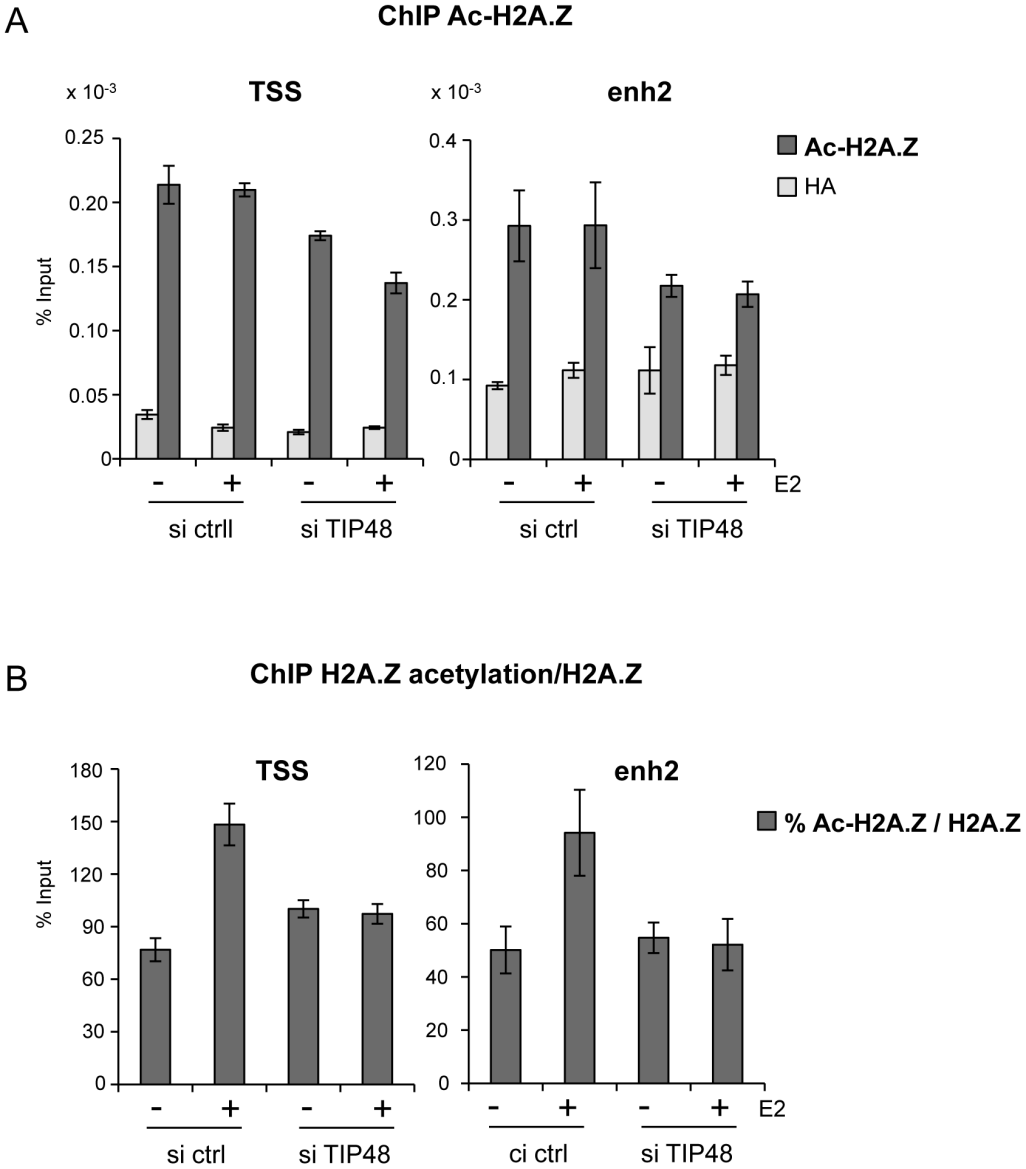
TIP48 promotes acetylation of H2A.Z. MCF-7 cells were cultivated and transfected as in Figure 2. A) Association of acetyl H2A.Z with *CCND1* TSS and enh2 sequences analyzed by ChIP and shown as % input (n = 2). B) Ratio of acetylated versus total H2A.Z in %.

TIP60 is found in protein complexes able to acetylate histones, with a preference for lysine 5 of H2A [36]. Core histones are generally acetylated in the promoter region of transcribed genes. Acetylation of the histone variant H2A.Z was shown to characterize active genes in yeast and recently also in prostate cancer cells [13], [37]. Using an antibody that specifically recognizes H2A.Z acetylated at 3 N-terminal lysines, we determined that a large fraction of H2A.Z bound to the *CCND1* promoter and to the 3′ enh2, was highly acetylated (Figure 5A). Acetylation levels of H2A.Z did not vary following E2 induced *CCND1* gene activation in control samples (Figure 5A). However, because H2A.Z was released during transcription activation, the ratio of acetylated H2A.Z/total H2A.Z increased nearly 2-fold at these sites (Figure 5B). In siTIP48 transfected cells, we observed a decrease in acetylated H2A.Z present at the TSS and the enh2 (Figure 5A). The increased ratio of acetylated H2A.Z associated with the *CCND1* gene following E2 was abolished in cells transfected with siTIP48 (Figure 5B). The reduced ratio of H2A.Z acetylation thus correlated with impeded transcription activation in siTIP48 transfected MCF-7 cells (Figure 5B and Figure 2C).

In conclusion, failure of TIP60 to associate with *CCND1* in the absence of TIP48 correlated with reduced binding of ERα (Figure 3), reduced levels of H2A.Z acetylation at the *CCND1* gene (Figure 5) and the inability to activate this gene by estrogen (Figure 2C).

### TIP48 modulates *CCND1* chromatin structure by controlling gene looping

Long-range chromatin interactions between ERa recognition sequences and enhancers have been proposed to regulate ERa-target genes in breast cancer cells [38], [39]. The main enhancer regulating *CCND1* is located at the 3′ end of the gene, 14 kb distant from the promoter [7]. Gene looping via promoter-enhancer crosstalk is associated with repressed, low *CCND1* expression in ERa-negative, MDA-MB231 cells [40]. Thus we asked whether this loop also existed in MCF-7 cells and more importantly, whether looping was sensitive to hormone.

**Figure 6:**
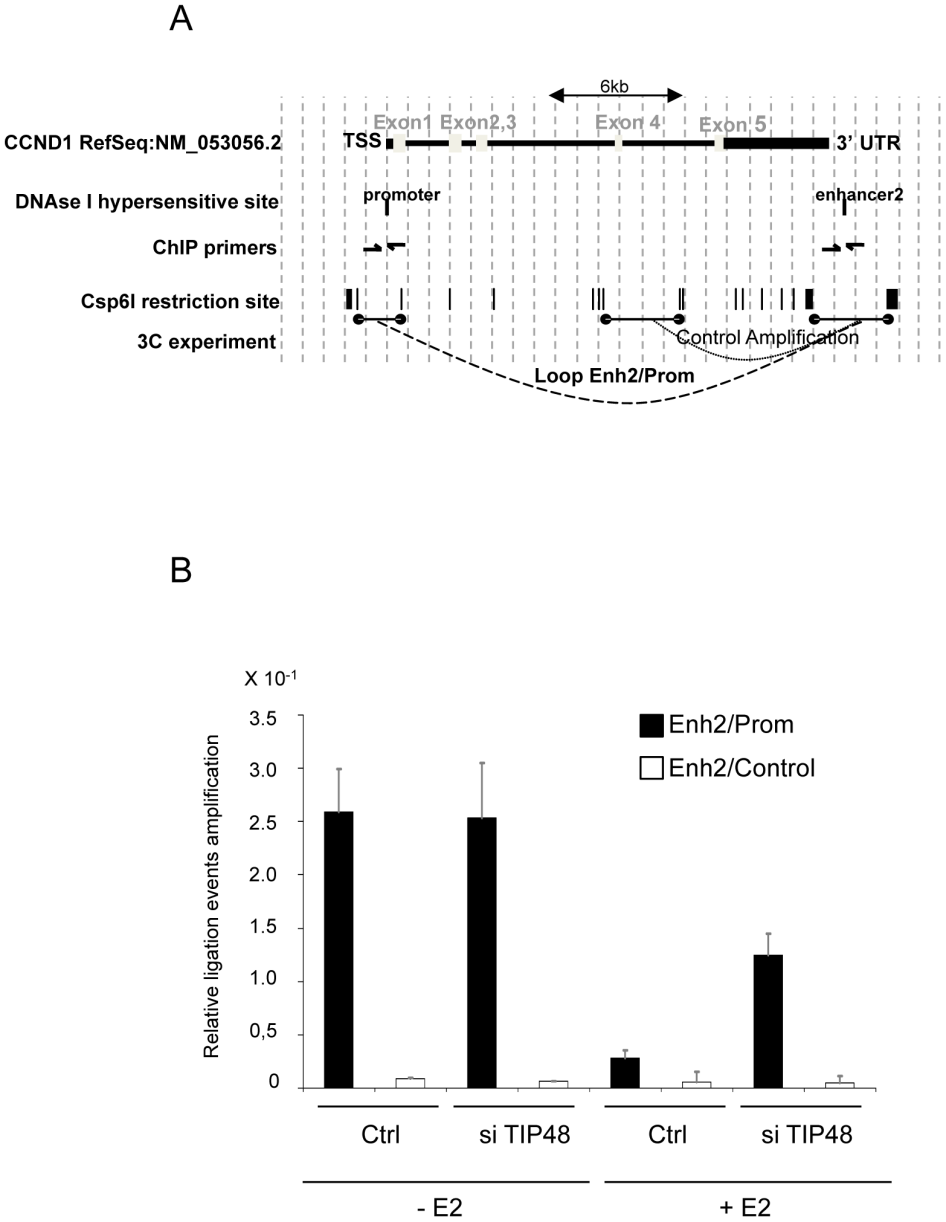
Release of *CCND1* gene looping requires TIP48. A) Schematic representation of the *CCND1* locus on h.s. chromosome 11 (adapted from the UCSS genome browser http://genome.ucsc.edu/cgi-bin/hgGateway). DNAseI hypersensitive sites [7], Csp6I restriction sites and fragments assayed by 3C are represented. B) Quantitative PCR amplification of ligation events in a 3C assay in MCF-7 cells treated or not with E2 10^−7^ M for 45 min and transfected 72 h before with scramble (ctrl) or TIP48 siRNA. Crosslinked DNA was digested with the Csp6I restriction enzyme and ligated. qRT-PCR was performed with primers designed for the 4 possible ligation events (only one is shown).

We used a chromatin conformation capture (3C) assay. The 3C method detects physical proximity between distal DNA sites by ligation of cross-linked restricted DNA fragments [41], [42]. Ligation products between enh2 and promoter, and between enh2 and a control fragment inside the *CCND1* ORF were amplified and normalized to an amplified enh2 PCR product (see Materials and Methods) (Figure 6A). We measured significant interaction frequencies between enh2 and promoter sequences in MCF-7 cells grown in hormone-stripped media (-E2). Interaction frequencies were reduced ∼10-fold 45 min after addition of E2 to the cell culture (Figure 6B). No significant amplification of ligation products between enh2 and the internal control fragment was detectable. Hence, an extragenic loop mediated by specific promoter enhancer interactions was present when *CCND1* expression is low (Figure 6B and Figure 2D). Upon transcription activation, gene looping is markedly reduced.

It was tempting to speculate that TIP48 plays a role in regulating looping. We assessed the relative frequencies of interaction between enh2/promoter and enh2/internal control fragments in MCF-7 cells transfected or not by siTIP48. Depletion of TIP48 had no impact on enh2/promoter contacts in the absence of E2 (Figure 6B). This observation correlated with identical basal expression levels of *CCND1* in control and siTIP48 transfected cells (Figure 2C).

**Figure 7:**
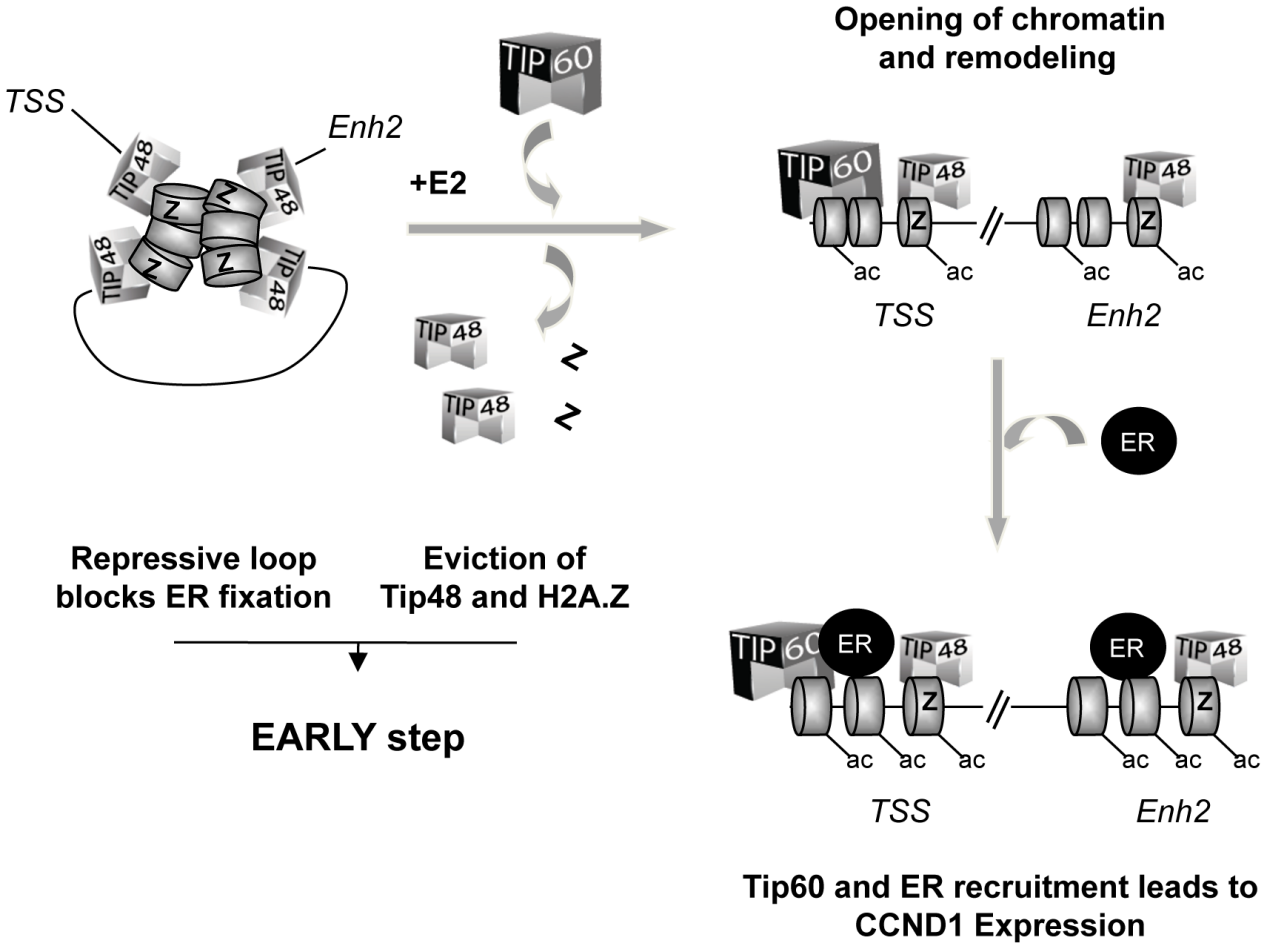
Model for the role of TIP48, H2A.Z, and TIP60 in initiation of transcription activation of *CCND1* via promoter enhancer crosstalk.

45 min after addition of E2 to the cells, the frequency of enh2/promoter interaction was 5-fold greater in siTIP48 transfected cells compared to control cells (Figure 6B). Conservation of significant repressive gene looping could thus account for impeded E2 bound ERa binding to the *CCND1* promoter and compromised transcription activation. We propose a model (Figure 7) in which TIP48 is required at early steps during transcription activation which is initiated by release of H2A.Z and subsequent dissociation of the enhancer from the promoter. E2 bound estrogen receptor can then recognize the promoter and stimulate transcription of *CCND1*.

## Discussion

We unraveled a role for TIP48 in initiating transcription activation of the *CCND1* oncogene. Recruitment of the histone acetyltransferase TIP60 is dependent on TIP48 and H2A.Z binding to the promoter and 3′ enhancer of the *CCND1* gene. We propose that low levels of *CCND1* expression are regulated because the associated gene loop is transcription-dependent. This regulation is brought about by the activity of TIP48 containing complexes which locally act upon chromatin structure to release a disabling loop. Such a mechanism allows fine-tuning transcription regulation of genes pivotal for the cellular equilibrium in rapidly changing environments.

Our work describes early events implicated in E2 induction of *CCND1*. These events include dynamic exchange of a series of cofactors, namely the TIP48 complex and histone variant H2A.Z, recruitment of TIP60 and acetylation of H2A.Z enabling the main transcription factor, the estrogen receptor, to associate with its target sequences. TIP60 can directly interact with ERa and its acetyltransferase activity is important during transcription initiation once ERa is bound to target gene promoters [34].

TIP60 is a versatile enzyme that functions with a variety of partners in a gene and cell specific manner [34], [43]. Selective knock-down of TIP60 by siRNA compromises activation of some, but not all ERa target genes in MCF-7 cells, as well as nuclear receptor independent genes in several cell lines (unpublished observations). *CCND1* was one of the genes found to be insensitive to siTIP60 [34]. This observation denotes that TIP60 can be replaced by other histone acetyltransferases in *CCND1* transcription activation. Thus, dependency of early chromatin remodeling steps on TIP48 and H2A.Z may be more generally applicable to allow cofactor recruitment for productive ERa binding in stimulated transcription.

We found that H2A.Z was removed from *CCND1* regulatory elements while this variant had previously been shown to be recruited to the promoter of the *TFF1* gene upon E2 treatment of MCF-7 cells [24]. Differences in promoter structure are a plausible explanation for divergent remodeling mechanisms. It is also likely that post-translational modifications of H2A.Z are important as shown in a recent genome wide study by Valdes-Mora *et al.* who found that H2A.Z acetylation at the TSS correlates with active transcription in prostate cancer cells [13]. Indeed, the level of acetylation of H2A.Z near the TSS of *CCND1* was equivalent at non-activated and E2 stimulated cells. We propose that, in ERα-positive breast cancer cells, the ratio of acetylated H2A.Z/H2A.Z rather than the total amount of H2A.Z bound to the *CCND1* promoter correlates with transcriptional activity.

Chromatin remodeling events are crucial for hormone stimulated activation of estrogen receptor target genes. However, so far, all data available describe the recruitment of remodeling complexes and cofactors once the estrogen receptor is bound. The Brg1 subunit of the SWI/SNF complex is one of the first proteins to associate with ERa and, although transcription is no longer activated in its absence, ERa remains bound in siBrg1 transfected cells [34]. Here we demonstrate that chromatin remodeling events prior to ERa binding are essential for initiating transcription. These events depend on TIP48 and H2A.Z specific nucleosome conformation. Chromatin structure impedes ERa loading via intragenic looping. Notably, interaction between promoter and enhancer sequences forms a repressive complex. Reduced distances between 5′ and 3′ ends of gene loci have been attributed to greater chromatin density. In this case, looping does not require changes in chromatin compaction. Dynamic release of gene loops is consistent with rapid chromatin remodeling and transcription activation by hormone.

Finally, addition of hormone triggers large scale chromatin remodeling. In breast cancer cells gene response to progestin is mediated by nucleosomes [44] and estradiol treatment leads to expansion of chromosome territories within minutes [45]. This latter phenomenon was also observed in ERα-negative cells (unpublished) suggesting that chromatin decondensation is independent of the receptor and may prepare its binding in ERα-positive cells. It is thus tempting to speculate that the signaling mechanism by which hormone addition primes chromatin triggers histone exchange and remodeling prior to ERα binding.

## Materials and Methods

### Cell lines, transfection, and Western blotting

MCF-7 cells were purchased from ATCC and were maintained in DMEM/F12 without phenol red with Glutamax containing 50 mg/ml gentamicin, 1 mM sodium pyruvate and 10% heat-inactivated and steroid free fetal calf serum (FCS) (Invitrogen). MCF-7 cells were treated with 10^−7^ M estrogen E2 (Sigma) for the indicated times. 5×10^6^ MCF-7 cells were transfected with 20 nM of H2A.Z siRNA ON-TARGET plus SMARTpool, TIP48 siRNA ON-TARGET plus SMARTpool or scrambled (scr) siRNA (Dharmacon Thermo Scientific) using Interferine (Ozyme). Cells were mock-transfected (pcDNA3.1) or transfected with 1 µg of pcDNA3.1/*TIP60* (gift from Dr. Didier Trouche) using the Amaxa Cell line Nucleofactor Kit V program P-020 according to the manufacturer's protocol. TIP60 siRNA [43] was purchased from Eurogentec, and transfected using Interferine (Ozyme). 5×10^5^ MCF-7 cells were seeded in 6 well plates. 72 h following siRNA transfection, total cell extracts were isolated and protein levels of H2A.Z, TIP48 and TIP60 analyzed by immunoblotting on gel SDS-page 15% using antibodies against H2A.Z (ABCAM, ab4174), TIP48 (gift of Dr. Mikhaïl Grigoriev) TIP60 [43] or GAPDH (Millipore, mab374).

### RNA analysis

Total RNA was extracted using an RNeasy mini-kit (Qiagen) and eluted with 35 µl of RNAase-free water. First strand cDNA was generated using 2 µg of total RNA in a reaction containing random oligonucleotides as primers with the ThermoScript RT-PCR system (Invitrogen). Real-time PCR was performed on a Mastercycler ep *realplex* ^4^ (Eppendorf) using the platinum SYBR Green q-PCR SuperMix (Invitrogen) according to the manufacturer's instructions. Amplification conditions: 1 min at 50°C, 3 min at 95°C followed by 40 cycles (20 s at 95°C, 20 s at 60°C, 20 s at 72°C). mRNA expression were normalized against expression levels of the *RPLP0* ribosomal gene used as an internal control. qRT-PCR primers: *H2AFZ*: 5′-CCTTTTCTCTGCCTTGCTTG-3′ and 5′-CGGTGAGGTACTCCAGGATG-3′, *CCND1*: 5′-GCGTCCATGCGGAAGATC-3′ and 5′-ATGGCCAGCGGGAAGAC-3′, *RPLP0*: 5′-TGGCAGCATCTACAACCCTGAA-3′ and 5′- CACTGGCAACATTG CGGACA-3′, TIP48: 5′-TGAAGAGCACTACGAAGACGC-3′ and 5′-CCTTACTACCCAGCTC CTGA- 3′.

### ChIP assays

ChIP analyses were performed as described previously [46]. Samples were sonicated to generate DNA fragments <500 bp. Chromatin fragments were immunoprecipitated using antibodies against H2A.Z (ab4174, ABCAM), acetyl H2A.Z (ab18262, ABCAM), TIP48 (gift of Dr. Mikhaïl Grigoriev), ERα (sc-543, Santa Cruz), H3 (ab1791, ABCAM), TIP60 [47] or an irrelevant HA antibody (H6908, Sigma). The precipitated DNA was amplified by real-time PCR, with primer sets designed to amplify the promoter (TSS) and *enh2* enhancer regions of the *CCND1* gene (Figure 1B). qRT-PCR primers: *CCND1* (TSS): 5′-CGGGCTTTGATCTTTGCTTA-3′ and 5′-ACTCTGCTGCTCGCTGCTAC-3′, distal *CCND1* enhancer (enh2): 5′-CAGTTTGTCTTCCCGGGTTA-3′ and 5′- CATCCAGAGCAAACAGCAG-3′. All ChIP data are shown as percent input.

### 3C assays

3C assays were performed essentially as described [48], [49], with minor modifications. MCF-7 cells were treated with E2 10^−7^ M for 45 mn or transfected with a scrambled control siRNA, with TIP48 SMARTpool siRNA (Dharmacon Thermo Scientific), and cultured in phenol red-free DMEM containing 10% FBS-T for 72 h before cross-linking. The culture medium was removed, and cells were fixed with 1.5% formaldehyde for 10 min at room temperature. Cells were then washed twice with cold phosphate-buffered saline solution, and resuspended in ice-cold lysis buffer (10 mm Tris-HCl, pH 8.0, 10 mm NaCl, 0.2% Nonidet P-40, and protease inhibitor mixture). Nuclei were resuspended in 1 ml of Buffer B 1.2× buffer (MBI Fermentas) supplemented with SDS 0,3%. Triton X-100 1,8% was added to sequester the SDS and incubated for 1 h at 37°C. The cross-linked DNA was digested overnight with 400 units of restriction enzyme *Csp*6I (MBI Fermentas). The restriction enzyme was inactivated by incubation at 65°C for 20 min. The reactions were diluted with ligase buffer (50 mm Tris-HCl, pH 7.5, 10 mm MgCl_2_, 10 mm dithiothreitol, 1 mm ATP, and 25 µg/ml bovine serum albumin), supplemented with Triton X-100 (1% final concentration). The DNA was ligated using T4 DNA ligase (New England Biolabs, Ipswich, MA) overnight at 16°C and an additional 100 units for 2 h at 37°C. RNase was added for 30 min at 37°C, and samples were incubated with SDS overnight at 70°C to reverse the crosslink. The following day, samples were incubated for 2 h at 45°C with proteinase K, and the DNA was purified by phenol-chloroform extractions and ethanol precipitation. Interaction between chromatin domains was assessed by real-time-PCR amplification for each predicted ligation event [48], [50]. Primers have been designed on the digested BAC fragments, directly around the putative site of ligation for the four possibilities. BAC clones RP11-300ID (BACPAC Resources Center at Childrens Hospital Oakland Research Institute, Oakland, CA) containing the *CCND1* gene and downstream 160-kb region were used. 40 ug of BAC was digested by Csp6I overnight and ligated. This product was purified by phenol chloroform and precipitated in order to generate 3C control templates. PCR primer efficiency was measured by amplifying 0.01 to 50 ng of digested BAC product and also tested on a fixed amount (50 ng) of digested genomic DNA. All primers have an annealing temperature between 65 to 70°C and a product size around 150–300 bp. All primer combinations showed PCR efficiency between 90 and 100%. 3C assay results are presented as the average from three independent preparations of 3C DNA, followed by qPCR analysis in triplicate. qPCR for enh2 (PCR primers design inside the Csp6I restriction fragment enh2) was used as an internal control to verify ligation events. Non-digested sample and ligation between a control fragment and enh2 were also performed (data not shown). Primers used for one of the four ligation event tested: Enh2/Prom: 5′-CTGGGAGAGATGGAGCTGAG-3′ and 5′-GGTTTTGTTGGGGGTGTAGA-3′, Enh2/ctrl: 5′-AAGCTCTCCCACAACCCATT-3′ and 5′-GTCAGCCCCACTGTTGACTC-3′. Other primers available upon request.

## Supporting Information

Figure S1 Depletion of TIP48 and H2A.Z by siRNA. MCF-7 cells were cultivated 3 days in steroid free medium and then induced by E2 10^−7^ M for 6 h. A) TIP48 mRNA expression levels analyzed by qRT-PCR and TIP48 protein analyzed by immunoblotting in siTIP48 transfected compared to control cells. B) *H2AFZ* mRNA expression levels analyzed by qRT-PCR and H2A.Z protein analyzed by immunoblotting in siH2A.Z transfected compared to control cells.
(PDF)

Figure S2 TIP60 overexpression and *H2AFZ* gene expression. MCF-7 cells were cultivated 3 days in steroid free medium and then induced by E2 10^−7^ M for 6 h. A) *TIP60* gene expression analyzed by qRT-PCR. TIP60 protein expression analyzed by western-blotting. B) *H2AFZ* gene expression levels were analyzed by qRT-PCR in siTIP48 transfected compared to control cells. C) *H2AFZ* mRNA expression levels analyzed by qRT-PCR in siTIP48 transfected compared to control cells.
(PDF)
