# Supplementary figures and images for: TIP48/Reptin and H2A.Z Requirement for Initiating Chromatin Remodeling in Estrogen-Activated Transcription
Source: PLoS Genet. 2013 Apr 18;9(4):e1003387. doi: 10.1371/journal.pgen.1003387 (PMC3630088; doi:10.1371/journal.pgen.1003387)

A

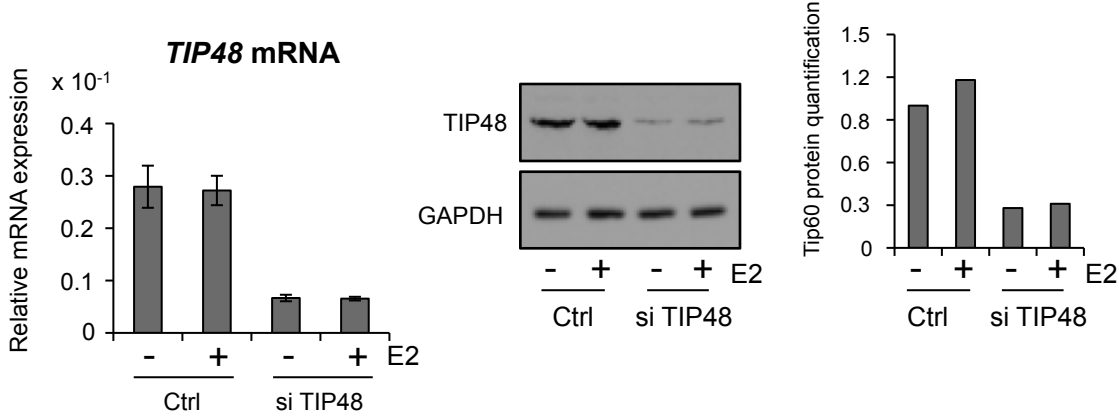

B

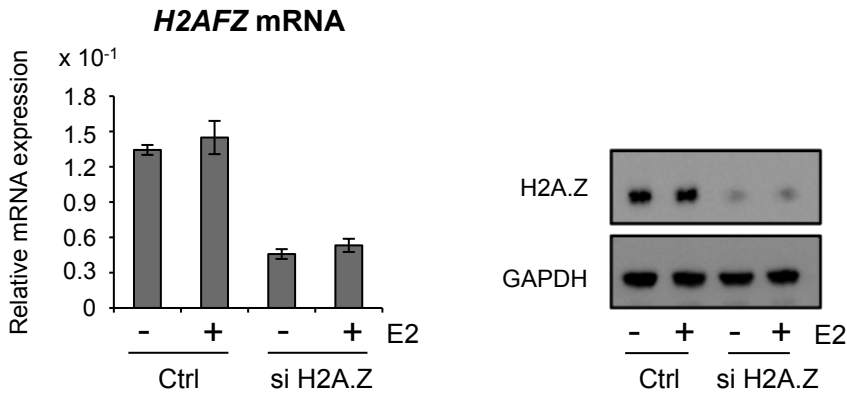

Supplement: Figure S1 — Depletion of TIP48 and H2A.Z by siRNA. MCF-7 cells were cultivated 3 days in steroid free medium and then induced by E2 10−7 M for 6 h. A) TIP48 mRNA expression levels analyzed by qRT-PCR and TIP48 protein analyzed by immunoblotting in siTIP48 transfected compared to control cells. B) H2AFZ mRNA expression levels analyzed by qRT-PCR and H2A.Z protein analyzed by immunoblotting in siH2A.Z transfected compared to control cells. (PDF) [file pgen.1003387.s001.pdf]

A

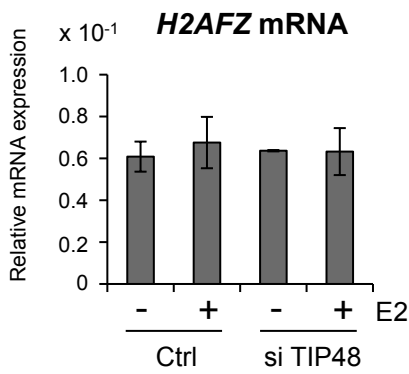

B

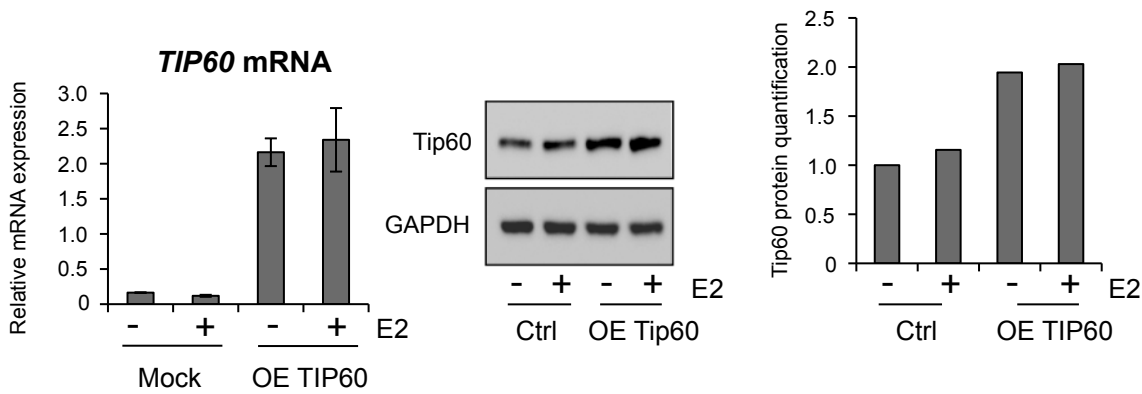

C

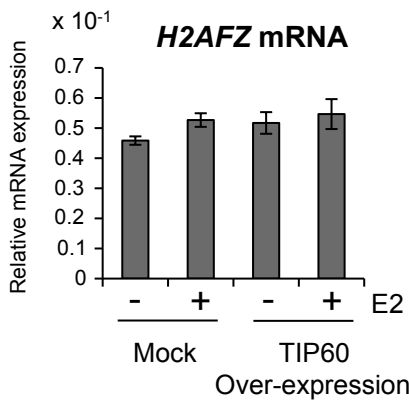

Supplement: Figure S2 — TIP60 overexpression and H2AFZ gene expression. MCF-7 cells were cultivated 3 days in steroid free medium and then induced by E2 10−7 M for 6 h. A) TIP60 gene expression analyzed by qRT-PCR. TIP60 protein expression analyzed by western-blotting. B) H2AFZ gene expression levels were analyzed by qRT-PCR in siTIP48 transfected compared to control cells. C) H2AFZ mRNA expression levels analyzed by qRT-PCR in siTIP48 transfected compared to control cells. (PDF) [file pgen.1003387.s002.pdf]
